# Whither Acid Rain?

**DOI:** 10.1100/tsw.2000.7

**Published:** 2001-04-04

**Authors:** Peter Brimblecombe

**Affiliations:** School of Environmental Sciences, University of East Anglia, Norwich, NR4 7TJ, UK

## Abstract

Acid rain, the environmental *cause célèbre* of the 1980s seems to have vanished from popular conscience. By contrast, scientific research, despite funding difficulties, has continued to produce hundreds of research papers each year. Studies of acid rain taught much about precipitation chemistry, the behaviour of snow packs, long-range transport of pollutants and new issues in the biology of fish and forested ecosystems. There is now evidence of a shift away from research in precipitation and sulfur chemistry, but an impressive theoretical base remains as a legacy.

The growth of scientific understanding and government regulation may explain a lack of media attention. Government agencies may look more at urban air pollution, which was neglected during the 1980s, or global issues such as the greenhouse effect or ozone hole.

It is easy for politicians to take the position that the acid rain problem has gone away. Emissions are lower, with a shift from high sulfur fuels, most particularly coal. In parallel the amount of sulfur deposited in rain Europe and North America has declined. Indeed, the decline in some parts of the UK and Germany has been so large that crops such as oats and oil seed rape can suffer from sulfur deficiency. Sulfur is now regularly added as a nutrient to soils in these regions.

However, the decreases in deposited sulfur are not matched by equivalent improvements in the amount of acid brought down in rain. One reason for this is that lower sulfur emissions have not always been accompanied by lower emissions of the nitrogen oxides, which give rise to atmospheric nitric acid. There has been a substantial rise in the N/S ratio in rainfall from many parts of Europe and North America. This nitrogen comes from large combustion sources (industry and power generation) and increasingly, significantly, the automobile. Furthermore, nitric acid is formed in the atmosphere through homogeneous processes, e.g., oxidation by ozone and the hydroxide radical. This contrasts with the oxidation of sulfur dioxide, a predominantly heterogeneous process, occurring in cloud and rain droplets. Thus the rate of formation and deposition of nitric acid is quite different from that found for sulfuric acid. The transition to acid rain dominated by nitrogen changes the distribution of acidity.

Another reason for smaller than expected improvements in rainfall acidity seem to be a decline in the concentration of alkalis in the atmosphere. Calcium was once more abundant and hence available to neutralise some of the acidity. In recent decades, the amount of alkaline particulate material has declined, perhaps because there is less dust from unsealed roads and less grit from industry and power generation.

On a broader scale, acid rain has moved with sulfur emissions to the rapidly industrializing world, most importantly the Asia-Pacific rim, where vast quantities of coal are now burnt. Research and regulation will need to confront a different acid rain problem here. Entirely new ecosystems will be confronted by acidic deposition. Air chemistry may be affected by the presence of alkaline Kosa dust. The greater extension of acid rain into the tropics, where soils are often deeply weathered, makes available new routes for mobilizing metals. Forest fires have raged in recent years, and although their contribution to the acid balance is unclear, they add to ozone concentrations and oxidation potentials. These emergent issues are every bit as important as the issues faced in the 1980s.

As with many environmental problems, policy makers everywhere need to remember that acid rain has not vanished, it is just the focus that has shifted.

Previously posted in NewsLab, TheScientificWorld.
© 2000 with author.
